# Visual Evoked Potentials Used to Evaluate a Commercially Available Superabsorbent Polymer as a Cheap and Efficient Material for Preparation-Free Electrodes for Recording Electrical Potentials of the Human Visual Cortex

**DOI:** 10.3390/s19224890

**Published:** 2019-11-09

**Authors:** Torsten Straßer, Susanne Kramer, Melanie Kempf, Tobias Peters, Anne Kurtenbach, Eberhart Zrenner

**Affiliations:** 1 Institute for Ophthalmic Research, Centre for Ophthalmology, University of Tuebingen, 72076 Tuebingen, Germany; tobias.peters@stz-eyetrial.de (T.P.); anne.kurtenbach@uni-tuebingen.de (A.K.); 2 University Eye Hospital, Centre for Ophthalmology, University of Tuebingen, 72076 Tuebingen, Germany; susanne.kramer@med.uni-tuebingen.de (S.K.); Melanie.Kempf@med.uni-tuebingen.de (M.K.); 3 STC Eyetrial at the Centre for Ophthalmology, 72076 Tuebingen, Germany; 4 Werner Reichardt Centre for Integrative Neuroscience (CIN), University of Tuebingen, 72076 Tuebingen, Germany

**Keywords:** biomedical electrodes, brain–computer interfaces, visual electrophysiology, superabsorbent polymer gel

## Abstract

The aim of this study was to investigate the use of inexpensive and easy-to-use hydrogel “marble” electrodes for the recording of electrical potentials of the human visual cortex using visual evoked potentials (VEPs) as example. Top hat-shaped holders for the marble electrodes were developed with an electrode cap to acquire the signals. In 12 healthy volunteers, we compared the VEPs obtained with conventional gold-cup electrodes to those obtained with marble electrodes. Checkerboards of two check sizes—0.8° and 0.25°—were presented. Despite the higher impedance of the marble electrodes, the line noise could be completely removed by averaging 64 single traces, and VEPs could be recorded. Linear mixed-effect models using electrode type, stimulus, and recording duration revealed a statistically significant effect of the electrode type on only VEP N75 peak latency (mean ± SEM: 1.0 ± 1.2 ms) and amplitude (mean ± SEM: 0.8 ± 0.9 µV) The mean amplitudes of the delta, theta, alpha, beta, and gamma frequency bands of marble electrodes were statistically significantly different and, on average, 25% higher than those of gold-cup electrodes. However, the mean amplitudes showed a statistically significant strong correlation (Pearson’s *r* = 0.8). We therefore demonstrate the potential of the inexpensive and efficient hydrogel electrode to replace conventional gold-cup electrodes for the recording of VEPs and possibly other recordings from the human cortex.

## 1. Introduction

Visual evoked potentials (VEPs) are changes of the electrical potential elicited by visual stimuli and recorded using electrodes mounted on the forehead and the scalp above the inion. VEPs are part of electroencephalogram (EEG) and are extracted by stimulus correlation and averaging. VEPs are used to measure the functional integrity of the visual pathways from retina via the optic nerves to the visual cortex [1]. A typical VEP waveform using pattern-reversal stimulation consists of a negative peak at about 75 ms (N75), followed by a positive peak at about 100 ms (P100) [2]. P100 is the standard measure of VEP analysis, which shows relatively little within-subject (intraocular) and between-subject variation [2]. Any abnormality that affects the visual pathway or visual cortex, such as optic neuritis, meningitis, stroke, tumors, or multiple sclerosis, can alter the VEP waveform by a reduced amplitude, a delayed latency, or a combination of both [1].

Recording of visual evoked potentials is usually carried out using gold-cup electrodes. To obtain a good signal-to-noise ratio [3], extensive preparation of the patient is required, including cleansing and eventually abrasion of the skin. Studies have shown that the main source of impedance is the epidermis, which is composed of dead dry cells [4]. The preparation process is uncomfortable for the patient, and removal of the gel residues after the recording often requires washing of the hair [5]. Additionally, abrasion of the skin creates a potential risk of infections [6].

The aim of this study was to explore the use of “water beads”, a commercially available hydrogel formulation that is also known as marble electrodes, for the recording of VEPs. The hydrogel, made of an acrylic sodium salt of cross-linked polyacrylic acid, is superabsorbent and inexpensive, and its use as an electrode avoids the lengthy preparation normally involved in VEP recordings. A comprehensive review of superabsorbent polymers, their properties, and their applications has been given by several authors (see e.g., [7,8,9,10,11,12]).

Because of its conductive and elastic qualities, hydrogel-based electrodes have wide use in electrical stimulation and recording, both in vivo and in vitro. Hydrogel polymer electrodes can be used in a range of forms [8,13,14,15] and have been shown to be important for monitoring health in human/machine interfaces [16,17,18,19]. Their use as electrodes for recording EEG has also been widely reported [5,20,21,22,23].

Here, we adapted established gold-cup electrodes for use with hydrogel water beads in an easy-to-use electrode cap for use in the clinic to record VEP. We evaluated the usability of the marble electrode and examined the effect of high impedance on amplitudes and peak times of VEPs.

## 2. Materials and Methods

### 2.1. Electrodes

**Figure 1:**
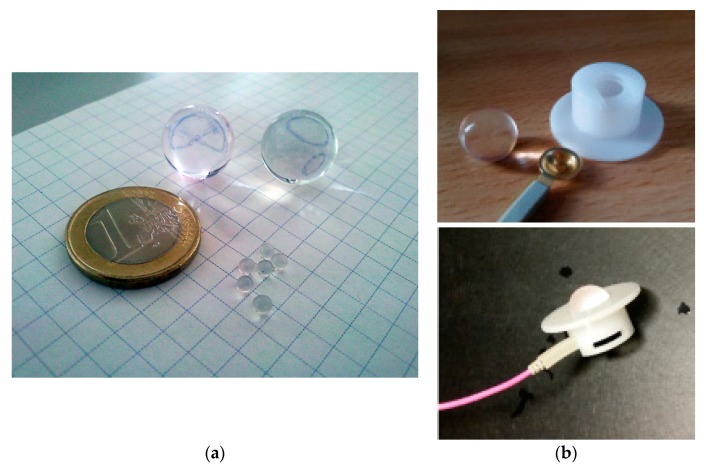
(**a**) Water beads before and after soaking for several hours in water. When fully swollen, the marble electrodes consist of up to 99.9% water and therefore become electrically conductive. (**b**) Top hat-shaped holder manufactured in a workshop of the University Eye Hospital, Tuebingen, and a marble electrode. The holders allow the marble electrode to be mounted at the scalp, while the connection between the marble and the amplifier is realized using a conventional gold-cup electrode. Top hat-shaped holders and gold-cup skin electrodes can be reused; the marble electrode is disposed of after use.

Water beads are made of an acrylic sodium salt of cross-linked polyacrylic acid (PAA) ([-CH_2_-CH(CO_2_Na)-]_n_), a hydrogel, which is able to absorb water up to 500 times its weight. After swelling, the beads consist of up to 99.9% water (Figure 1a), rendering them electrically conductive. The conductance of a marble electrode with a diameter of 1 cm is about 1250 µS (0.8 kΩ, measured with the Diagnosys Espion e^2^, 500 nA at 50 Hz) and therefore similar to that of tap water. Water beads are commonly used for watering plants or for decoration purposes and are available in home improvement stores and garden centers as well as online for about €2 per 1000 pieces. The marble electrodes are always damp but, in contrast to a sponge, they do not loose water when squeezed. The stiffness of a marble electrode is about κ = 0.4 N/mm.

**Figure 2:**
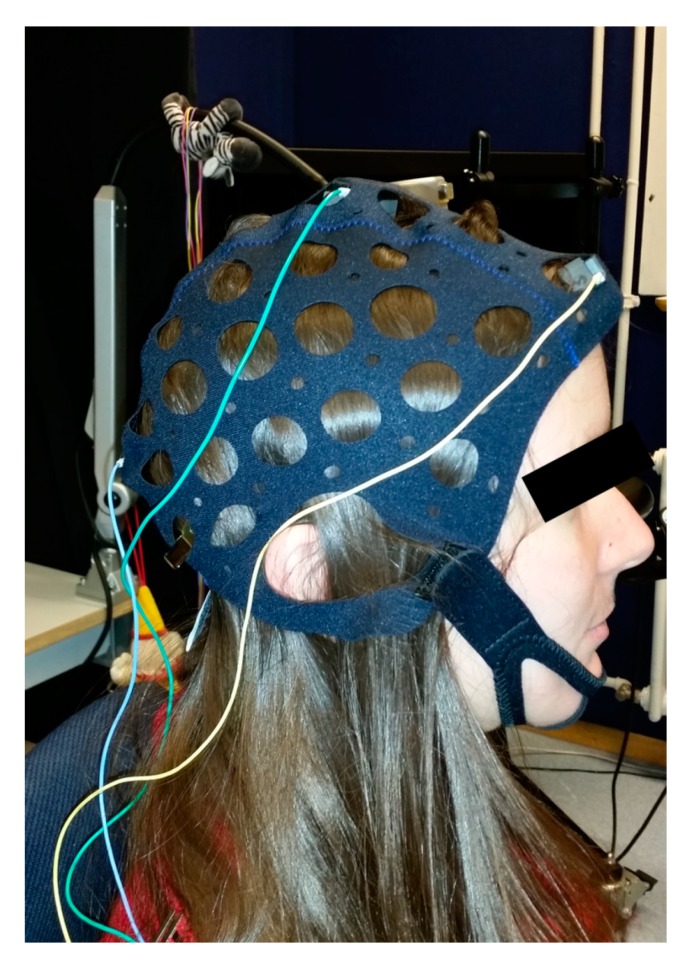
Participant prepared for visual evoked potential (VEP) recording using marble electrodes. The electrodes were mounted according to the International Society for Clinical Electrophysiology of Vision (ISCEV) recommendations at *Oz*, *Fz*, and *Cz* using an electrode cap with cup holders and marble electrodes. No scalp abrasion was done.

Top hat-shaped holders for the marble electrodes were manufactured from plastic (Perspex) in a workshop of the University Eye Hospital, Tuebingen. The inner diameter of the top hat corresponds to the diameter of the marble electrodes, and its height is about half the diameter of the marble electrode. A conventional gold-cup electrode clipped into the upper end of the hat (opposite to the brim) connects the leads with the marble electrode (Figure 1b). These holders allow the marble electrodes to be positioned on the scalp using a commercially available electrode cap (Figure 2). When mounted on the head, the marble electrode is pressed at the same time onto the skin and the gold-cup electrode, ensuring a tight contact.

The holder and the skin electrode can be reused, while the marble electrode can be disposed of after use. The marble electrodes were soaked in pure water for about six hours, until they were swollen to their maximal size of about 1 cm in diameter.

### 2.2. Participants

Twelve healthy volunteers (nine female, three male; age 22–54 years, mean 36.6 years) with best-corrected visual acuity and no history of eye or neurological diseases were recruited from the staff of the Centre for Ophthalmology of the University of Tuebingen. All volunteers gave informed consent. The study followed the tenets of the Declaration of Helsinki and was approved by the Institutional Review Board of the Faculty of Medicine, University of Tuebingen.

### 2.3. Visual Stimulation

The VEP recordings were performed monocularly with one eye covered with an eye patch. The checkerboard stimulus was presented using a 21” CRT monitor (Model V999, Elonex, Birmingham, UK) [24] at a distance of 150 cm. Checkerboards of two check sizes—0.84° and 0.25°—were presented with a contrast of 80% and two reversals per second, according to the International Society for Clinical Electrophysiology of Vision (ISCEV) guidelines [2]. Each stimulus was presented three times, resulting in a total stimulation time of 2 × 61 s.

### 2.4. Data Acquisition

Electrodes were mounted according to the International 10–20 system [2,25]: active electrode above the inion at *Oz*, reference electrode at *Fz*, and ground electrode at *Cz*.

In the first session, marble electrodes were used as active, reference, and ground electrodes. No skin abrasion was performed. In the subsequent second session, the skin was cleaned using abrasive paste, and gold-cup electrodes were applied using conductive paste.

VEPs were recorded using an Espion e^2^ (Diagnosys Ltd., Cambridge, UK) with a sampling frequency of 1000 Hz and digitally band-pass filtered (1.25–100 Hz). No notch filter was used. Post-trigger time was 300 ms. Automated baseline correction was applied by averaging and subtracting a 20 ms pretrigger period. Three averages, consisting of 64 single sweeps, were recorded for each check size [2].

Cursor positions for N75 and P100 [2] were determined automatically as maximum or minimum value, respectively, within the expected time frames using the built-in peak-finding algorithm of the Espion acquisition software and manually adjusted if necessary. Peak times and amplitudes of N75 and P100 were exported for further analysis using a custom-developed software [26,27].

The impedance between the electrodes mounted at *Cz* (ground electrode) and *Oz* (active electrode) and at *Cz* and *Fz* (reference electrode) was measured before the start and after the end of the recording for either the marble electrodes or the gold-cup electrodes using the Espion acquisition software. Because the top hat-shaped holders contain a gold-cup electrode for connecting the marble electrode with the amplifier, the following components contribute to the impedance measurement for the marble electrodes: gold-cup electrode–marble electrode–skin–marble electrode–gold-cup electrode, while the following components contribute to the impedance measurement for the gold-cup electrodes: gold-cup electrode–skin–gold-cup electrode.

### 2.5. Signal Processing

**Figure 3:**
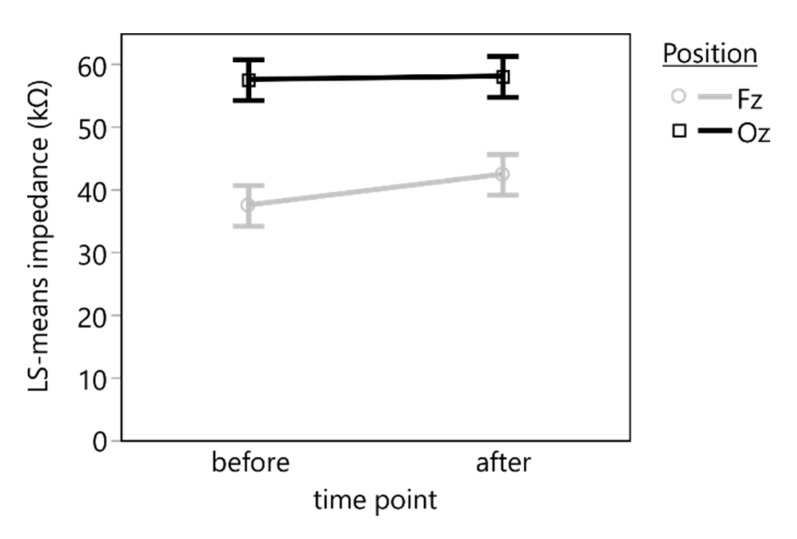
Least square means (LS means) and standard errors of the impedance of the marble electrodes measured before and after the VEP recording. A statistically significant difference in the impedance was found between the electrode positions *Fz* and *Oz* but not during the time between the recordings.

As the Espion acquisition software automatically stores the recordings as event-related potentials, the 384 sweeps (2 stimuli × 3 averages × 64 sweeps), each of 320 ms duration, were concatenated and corrected for the automatic baseline removal to reconstruct the original traces with duration of 122.88 s for each subject and electrode type (Figure 3). The traces were segmented into 2048 ms epochs and subjected to fast Fourier transformation (FFT) analysis with no windowing and ~0.5 Hz resolution. Mean amplitude values for delta (1.5–3.5 Hz), theta (4–7.5 Hz), alpha (8–12 Hz), beta 1 (13–16 Hz), beta 2 (13–21 Hz), beta 3 (21–32 Hz), and gamma (35–45 Hz) frequency bands were exported for statistical analysis [28].

### 2.6. Statistical Analysis

Linear mixed-effects models, fit by restricted maximum likelihood estimates (REML), were used to assess the significance of the electrode type in explaining variations in electrode impedance, mean amplitude of different frequency bands, and VEP N75 and P100 peak times and amplitudes. For all models, the variance inflation factors (VIF) of the predictors were calculated and assured to fall well below the common threshold value, indicating no collinearity between them. Prior to utilizing the results of the models, the normal distribution of the model residuals was confirmed visually, and the homoscedasticity of the variances of the residual was ensured using the Brown–Forsythe test and reported in case of violations.

To increase the statistical power of the analysis despite the small number of subjects, the alpha level was raised to 0.5 for all statistical tests, except otherwise stated.

All statistical analyses were carried out using JMP 14.2.0 (SAS Institute Inc., Cary, NC, USA).

#### 2.6.1. Electrode Impedance

A linear mixed-effects model (Equation (1)) was used to assess the effect of the recording duration on the impedance of the marble electrode (*Y*), with the position (*Fz*/*Oz*) (*α*) and the time point (before/after) (*β*) as well as their interaction set as categorical effects and the subject set as random effect (*ρ*).

(1) $$Y_{ijk}=\mu +\rho_{i}+\alpha_{j}+\beta_{k}+{\left(\alpha \beta \right)}_{jk}+\varepsilon_{ijk}$$

#### 2.6.2. Frequency Analysis

The correlation of the mean FFT amplitudes for delta, theta, alpha, beta 1, beta 2, beta 3, and gamma frequency bands were assessed by calculating the bivariate correlation coefficient between the conventional gold-cup electrode and marble electrode for each frequency band [28]. Additionally, a linear mixed-effects model (Equation (2)) was used to assess the effect of the electrode type on the log-transformed mean FFT amplitude value (*Y*) of the delta, theta, alpha, beta 1, beta 2, beta 3, and gamma frequency bands. The electrode type (*α*) and the frequency band (*β*) as well as their interaction were set as categorical effects, and the subject was set as random effect (*ρ*).

(2) $$Y_{ijk}=\mu +\rho_{i}+\alpha_{j}+\beta_{k}+{\left(\alpha \beta \right)}_{jk}+\varepsilon_{ijk}$$

#### 2.6.3. VEP Analysis

Linear mixed-effects models were used to assess the significance of electrode type and recording duration in explaining variations in the amplitudes and peak times of N75 and P100 (*Y*), respectively, with electrode type (*α*) and check size (*β*) set as categorical effects, recording duration (*γ*) set as continuous factor nested in check size, and subject (*ρ*) set as random effect (Equation (3)).

(3) $$Y_{ijk}=\mu +\rho_{i}+\alpha_{j}+\beta_{k}+\gamma_{l\left(k\right)}+{\left(\alpha \beta \right)}_{jk}+{\left(\alpha \gamma \right)}_{jl\left(k\right)}+\varepsilon_{ijk}$$

## 3. Results

### 3.1. Electrode Impedance

Compared to the conventional gold-cup electrodes, whose impedance was kept well below 5 k according to the ISCEV standard [29], the marble electrodes had far larger impedance, ranging from 20 to 80 kΩ.

The linear mixed-effects model (*n* = 40, *R*^2^ = 0.27) revealed a statistically significant effect of the electrode position (*F*(1, 27) = 13.8627, *p* = 0.0009), but neither the time point (*F*(1, 27) = 0.3245, *p* = 0.5736) nor the interaction between time point and electrode position (*F*(1, 27) = 0.2109, *p* = 0.6497) had a statistically significant effect on the electrode impedance (Figure 3).

A post hoc comparison using a *t*-test indicated a statistically significant mean difference of 17.9 (95% CI: [14.6, 21.2]) kΩ between *Oz* (mean: 57.7 kΩ) and *Fz* position (mean: 40.0 kΩ) (*t*(27) = 3.7233, *p* = 0.0009).

### 3.2. Frequency Analysis

**Figure 4:**
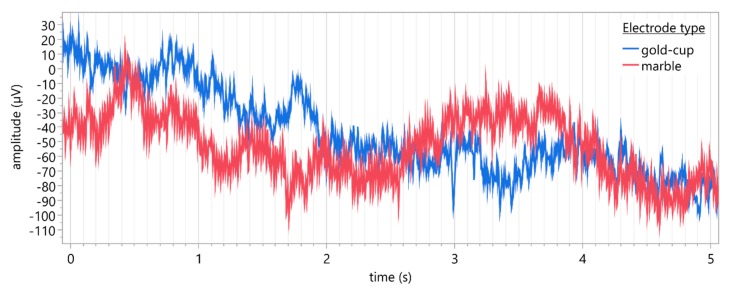
Representative five-second sample of reconstructed VEP traces (*Oz*: active, *Fz*: reference, *Cz*: ground) recorded using conventional gold-cup electrodes (blue) and marble electrodes (red) for a single participant. Sampling rate: 1000 Hz, filters: band-pass (1–100 Hz).

Figure 4 provides a five-second sample of reconstructed VEP traces taken from both conventional gold-cup (blue) and marble (red) electrodes for a single participant.

In the correlation analysis, statistically significant positive relationships (alpha level = 0.05) with large correlation coefficients between the conventional gold-cup and the marble electrode mean FFT amplitudes was observed for theta, alpha, beta 1, beta 2, beta 3, and gamma frequency bands as well as for the mains frequency (50 Hz). Table 1 provides a numeric summary of the Pearson’s *r*, the 95% confidence intervals, and significance values.

**Figure 5:**
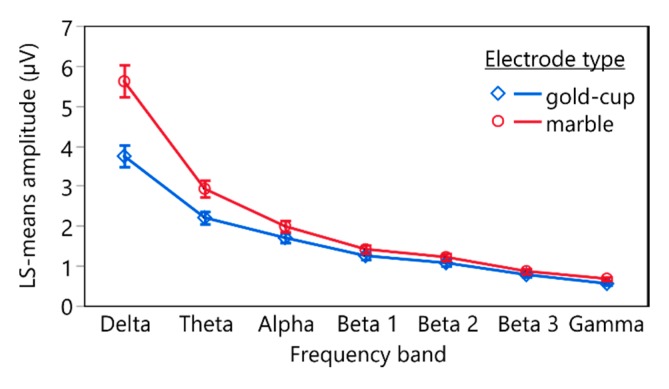
Estimated LS means and standard error (whiskers) of the delogarithmized means of the fast Fourier transformation (FFT) of the different frequency bands recorded using conventional gold-cup electrodes (blue) and marble electrodes (red).

The linear mixed-effects model (*n* = 168, *R*^2^ = 0.95) revealed statistically significant effects of the electrode type (*F*(1, 143) = 52.0405, *p* < 0.0001), the frequency band (*F*(6, 143) = 349.3580, *p* < 0.0001), and the interaction between electrode type and frequency band (*F*(6, 143) = 2.2029, *p* = 0.0460) on the log-transformed mean FFT amplitude. Figure 5 depicts the estimated least square means and the standard error of means (whiskers) of the FFT amplitudes of the different frequency bands recorded with conventional gold-cup electrodes (blue) and marble electrodes (red).

Post hoc comparisons using contrasts revealed statistically significant differences between FFT amplitudes recorded with conventional gold-cup electrodes and marble electrodes for all frequency bands. Table 2 lists the corresponding test statistics.

### 3.3. VEP Results

**Figure 6:**
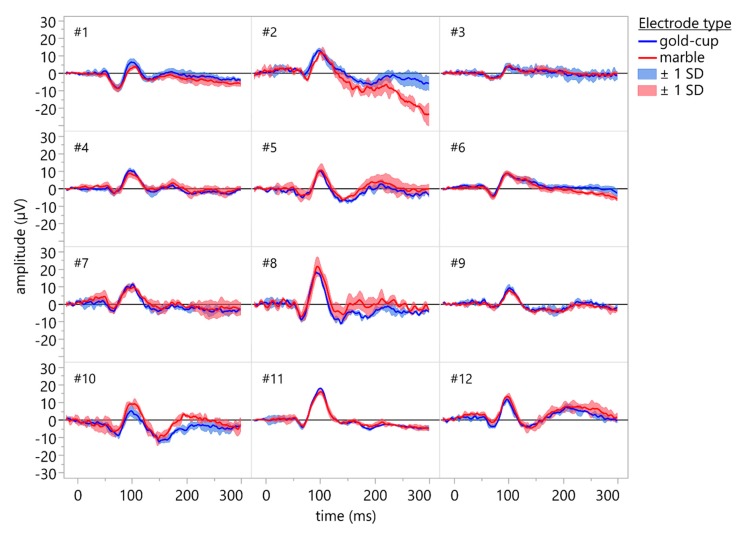
Individual VEP waveforms of the participants (grand average of 3 × 64 single sweeps). Recordings were done using conventional gold-cup electrodes (blue traces) and marble electrodes (red traces). Shaded areas indicate ±1 standard deviation. No cleansing or abrasion was used for the marble electrodes. Both electrode types resulted in comparable recordings, and the line noise was mostly eliminated through averaging.

VEPs could be recorded in all subjects using conventional gold-cup electrodes and marble electrodes. Figure 6 depicts the grand averages of 3 × 64 single traces for all subjects recorded using conventional gold-cup electrodes (blue traces) and marble electrodes (red traces). Both electrode types resulted in comparable recordings, and the line noise was mostly eliminated through averaging.

#### 3.3.1. Summary Statistics of N75 and P100 Peak Times and Amplitudes

Table 3 presents summary statistics for peak times and amplitudes of the N75 and P100 cursors recorded using gold-cup and marble electrodes.

#### 3.3.2. Effect of Electrode Type on N75 and P100 Peak Times and Amplitudes

Although the residuals of the linear mixed-effects models of N75 amplitude, N75 peak time, and P100 peak time were heteroscedastic, i.e., the variances were unequal, (*F*(1, 142) = 7.3829, *p* = 0.0074; *F*(1, 142) = 5.2284, *p* = 0.0237; *F*(1, 142) = 7.8444, *p* = 0.0058), the models were used for further analysis. Because the groups were balanced, the variance of the residuals did not depend on the electrode type [30], and the ratio of the maximum to the minimum variance between the groups was less than four for all models [31].

A statistically significant effect on the amplitudes or peak times of N75 and P100 was found for the check size used for stimulation in all models. The electrode type was found to have a statistically significant effect on the N75 amplitude and peak time as well as the interaction of electrode type and check size on the P100 amplitude and peak time. For the P100 amplitude, there was a statistically significant interaction between the electrode and check size. For the P100 peak time, there was a statistically significant interaction between the recording duration and check size (Table 4).

**Figure 7:**
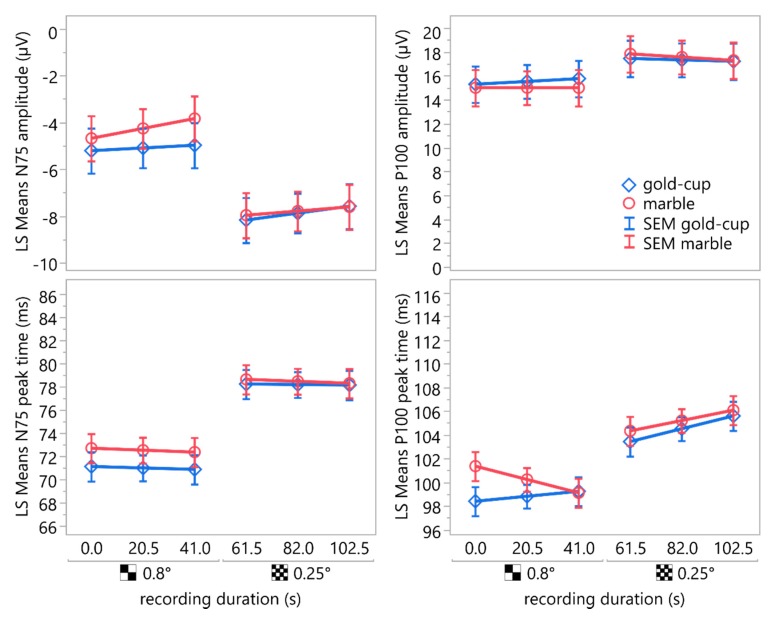
LS mean plots depicting the peak times and amplitudes of N75 and P100 acquired using gold-cup and marble electrodes using two different stimulus check sizes—0.8° and 0.25°—over the recording duration. The whiskers indicate the standard error of means. The means showed a statistically significant difference for the check size. The effect of the electrode type was statistically significant only for the N75 amplitude and peak time but not for P100. Neither the recording duration nor the interactions between electrode type and recording duration or check size were statistically significant.

Figure 7 shows the least square means of the peak times and amplitudes of N75 and P100 acquired using gold-cup and marble electrodes using the two different stimulus check sizes—0.8° and 0.25°—over the recording duration, along with their standard error of means.

Post hoc comparisons of least square means of the N75 amplitudes and peak times using the Student’s *t*-test indicated statistically significant differences between the marble electrode and the gold-cup electrode (amplitude: mean (marble) = −5.85 µV, mean (gold-cup) = −6.63 µV, SEM = 0.94, difference = 0.79 µV, 95% CI: [0.24, 1.31], *t*(125) = 0.9845, *p* = 0.3268; peak time: mean (marble) = 75.49 ms, mean (gold-cup) = 74.52 ms, SEM = 1.23, difference = 0.97 ms, 95% CI: [0.29, 1.64], *t*(125) = 0.9675, *p* = 0.3352).

## 4. Discussion

Visual evoked potentials were successfully recorded in 12 volunteers for two check sizes using marble electrodes and conventional gold-cup electrodes. The marble electrodes had about 10 times higher impedance compared to the gold-cup electrodes.

To reduce the impedance, we tried using saline solution instead of water for soaking the hydrogel in order to increase its electrical conductivity by adding Na^+^ and Cl^−^ ions. However, in the saline solution, the swelling ability was drastically reduced. This effect was also shown by Horkay et al. [32], who investigated the swelling properties of hydrogels in various physiological salt solutions. Furthermore, using saline solution did not result in decreased impedance (data not shown).

Even though ISCEV recommends keeping the electrode impedance lower than 5 kΩ [2,33], this is nowadays less justified because modern amplifiers have very high input impedance, up to gigaohms. The Diagnosys Espion e^2^ system used in this study has an input impedance of one gigaohm. A high impedance of the electrode may cause only problems when using old amplifiers with an input impedance of less than 100 megaohms. This is in line with the findings of Ferree et al. [6] and Kappenman and Luck [34], who found no significant difference between high impedance recordings and those with an impedance less than 5 kΩ. Furthermore, in the frequency range of interest, from 1 to 100 Hz, bioelectrical-generated noise dominates the recording and lowering the impedance by, e.g., skin abrasion, improves the signal-to-noise ratio only by a few percent [3].

The statistically significant lower impedance of the marble electrodes mounted on *Fz* compared to *Oz* is probably the result of a worse contact at *Oz*, caused by dense hair [21]. Combing the hair before mounting the electrodes may reduce this problem [35]. Furthermore, it is possible that the contact pressure at *Oz* may have been lower than at *Fz*, resulting in a smaller contact area of the marble electrode, which is related to an increase in noise [4].

The difference in the impedance between *Fz* and *Oz* is likely the reason for picking up of line noise, which caused a contamination of the single sweeps with a 50 Hz signal. Line noise usually results from electrodes with high impedance, as is the case with marble electrodes, in combination with a differential amplifier [4].

The increased noise caused by the higher impedance of the marble electrodes may have also led to the statistically significant higher mean amplitudes in the Fourier analysis of the different frequency bands of the recordings compared to those of conventional gold-cup electrodes. On average, the mean amplitudes recorded using gold-cup electrodes were between 67% and 82% lower than for marble electrodes. However, the mean amplitudes of the different frequency amplitudes obtained with marble electrodes showed a statistically significant high correlation to those recorded with conventional gold-cup electrodes.

Even though the single traces were strongly contaminated with line noise, these artifacts could be removed almost completely using averaging and therefore only had a small effect on the measured amplitudes and peak times of the VEP. Linear mixed-effect models revealed a good agreement between the cursors obtained from recordings of the marble electrodes and the conventional gold-cup electrodes. A statistically significant difference was found for the N75 amplitude and the peak time between recordings using marble electrodes and conventional gold-cup electrodes. For the P100 peak time and amplitude, the interaction between check size and electrode type showed a statistically significant effect. A statistically significant effect of the recording duration was only found as an interaction with the check size and with the electrode type for the P100 peak time. All effects, except check size, reached statistical significance only because the alpha level was raised to 0.5. Furthermore, the mean differences in amplitudes and peak times between the conventional gold-cup electrode and the marble electrode were in the range of the intrasubject variability published by several groups [36,37,38,39] and those reported by Tello et al. for the repeatability of transient visual evoked potentials [40] and therefore of no clinical relevance.

## 5. Conclusions

This study demonstrated the potential of marble electrodes to replace conventional gold-cup electrodes for the recording of visual evoked potentials. Using modern differential amplifiers and averaging, high-quality VEP recordings were obtained without scalp abrasion. The differences in the amplitudes and the peak times between conventional gold-cup and marble electrodes were within the range of intrasubject variability and therefore of no clinical relevance. The ease of use and their low cost may render marble electrodes useful for other application domains, such as simultaneous recordings of the electroencephalogram during functional magnetic resonance imaging, brain–computer interfaces, or transcorneal electrical stimulation. However, further studies are needed to evaluate such applications, especially with regard to the differences in the amplitudes of the Fourier spectrum in the different frequency bands.

In a previous study, we demonstrated the application of marble electrodes for the recording of electroretinograms in small animals (Strasser et al., IOVS2012, Vol. 53, 2462). Additional uses for the application of marble electrodes may be brain–computer interfaces. This is because, in contrast to currently used electrodes, hydrogel-based electrodes, such as marble electrodes, provide a higher wearing comfort and are better tolerated, as Pinegger et al. investigated [5].

As marble electrodes consist of up to 99% water, they do not dry out, rendering them useful for long-time recordings, e.g., for brain–computer interfaces or during functional magnetic resonance imaging.

Avoiding the need for cleansing and abrasion of the skin increases patient comfort and significantly reduces the time for preparation. Additionally, it eliminates the risk of infection during abrasion of the skin [6]. Because the marble electrode is disposed of after the recording, time-consuming disinfection or sterilization of the electrodes can be omitted as well.

Marble electrodes may also be used for transcorneal electrical stimulation [41]. Several companies provide commercial devices (e.g., the OkuStim system, Okuvision GmbH, Reutlingen, Germany), which usually use modified versions of Dawson–Trick–Litzkow (DTL) electrodes [42]. These could be replaced by sterile marble electrodes and therefore ease the application for patients.

## 6. Patents

T.S., T.P., and E.Z. hold a patent on using this type of hydrogel electrodes for electrophysiological applications (T. Strasser, “Grundkörper, Halter, Kit und Elektrodenanordnung sowie Verfahren zur Herstellung”, DE 10 2012 101 337 B4, issued October 31, 2013).

## Figures and Tables

**Table 1:** Average correlations of conventional gold-cup and marble electrode signals for each frequency band and the mains frequency (*n* = 12 subjects).

Frequency Band	Pearson’s *r*	95% CI	*p*-Value
Delta (1.5–3.5 Hz)	0.84	[0.51, 0.95]	0.0007 ***
Theta (4–7.5 Hz)	0.85	[0.54, 0.96]	0.0004 ***
Alpha (8–12 Hz)	0.92	[0.73, 0.98]	<0.0001 ***
Beta 1 (13–16 Hz)	0.75	[0.31, 0.93]	0.0048 **
Beta 2 (13–21 Hz)	0.80	[0.43, 0.94]	0.0016 **
Beta 3 (21–32 Hz)	0.71	[0.23, 0.91]	0.0094 **
Gamma (35–45 Hz)	0.62	[0.07, 0.88]	0.0319 *
Mains noise (50 Hz)	0.63	[0.09, 0.89]	0.0269 *

Note: Alpha level = 0.05. Stars indicate the level of significance: * *p* < 0.05, ** *p* < 0.01, *** *p* < 0.001.

**Table 2:** Results of the post hoc contrast tests comparing the mean FFT amplitude of the different frequency bands recorded using conventional gold-cup electrodes and marble electrodes. The difference in the log-transformed LS mean amplitudes was converted to amplitude ratio.

Frequency Band	LS Means Amplitude Ratio	*df_nom_*	*df_den_*	*p*-Value
Delta (1.5–3.5 Hz)	0.67	1	143	<0.0001 ***
Theta (4–7.5 Hz)	0.75	1	143	0.0002 ***
Alpha (8–12 Hz)	0.86	1	143	0.0347 **
Beta 1 (13–16 Hz)	0.88	1	143	0.0929 **
Beta 2 (13–21 Hz)	0.88	1	143	0.0878 **
Beta 3 (21–32 Hz)	0.90	1	143	0.1471 *
Gamma (35–45 Hz)	0.82	1	143	0.0081 ***

Note: Alpha = 0.5. *df_nom_* indicated degrees of freedom numerator. *df_den_* indicates degrees of freedom denominator. Stars indicate the level of significance: * *p* < 0.5, ** *p* < 0.1, *** *p* < 0.01.

**Table 3:** Summary statistics for peak times and amplitudes of N75 and P100 recorded using gold-cup and marble electrodes from 12 subjects. Each average consists of 64 single sweeps.

Check size	Cursor	Recording Duration (s)	Amplitude M ± SD (µV)		Peak Time M ± SD (ms)	
			Gold-Cup	Marble	Gold-Cup	Marble
0.8°	N75	0	−5.02 ± 3.27	−4.78 ± 2.52	71.42 ± 4.74	72.75 ± 5.75
		20.5	−5.50 ± 3.07	−4.08 ± 3.07	70.42 ± 4.08	72.42 ± 4.70
		41.0	−4.79 ± 3.07	−3.94 ± 2.41	71.17 ± 4.76	72.42 ± 5.63
	P100	0	15.37 ± 5.09	15.00 ± 5.83	97.83 ± 3.21	101.08 ± 4.23
		20.5	15.37 ± 5.48	14.99 ± 5.33	99.92 ± 2.31	100.75 ± 3.25
		41.0	15.84 ± 4.88	15.00 ± 6.47	98.67 ± 3.14	98.83 ± 4.37
0.25°	N75	61.5	−8.05 ± 3.94	−7.65 ± 3.94	78.33 ± 4.12	78.58 ± 2.64
		82.0	−8.13 ± 3.97	−8.45 ± 4.63	77.92 ± 3.85	78.50 ± 3.40
		102.5	−7.46 ± 3.36	−7.29 ± 3.70	78.25 ± 5.40	78.25 ± 4.16
	P100	61.5	17.21 ± 4.53	17.62 ± 6.29	103.75 ± 6.43	104.17 ± 3.88
		82.0	17.82 ± 4.77	17.98 ± 5.77	103.83 ± 5.10	105.50 ± 4.95
		102.5	16.97 ± 5.23	17.09 ± 5.01	105.92 ± 5.68	105.92 ± 5.38

Note: M and SD represent mean and standard deviation, respectively.

**Table 4:** Results of the linear mixed-effects models for peak times and amplitudes of N75 and P100.

Model	*R* ^2^_adj_	Effect	*df_nom_*	*df_den_*	*F*-Value	*p*-Value
N75 amplitude (*n* = 144)	0.66	Check size	1	125	24.4845	<0.0001 ***
		Electrode	1	125	0.9693	0.3268 *
		Duration (check size)	2	125	0.5974	0.5518
		Electrode × check size	1	125	0.4229	0.5167
		Duration × electrode (check size)	2	125	0.1254	0.8822
N75 peak time (*n* = 144)	0.75	Check size	1	125	48.0600	<0.0001 ***
		Electrode	1	125	0.9360	0.3352 *
		Duration (check size)	2	125	0.0941	0.9102
		Electrode × check size	1	125	0.2511	0.6171
		Duration × electrode (check size)	2	125	0.0127	0.9874
P100 amplitude (*n* = 144)	0.75	Check size	1	125	5.5562	0.0200 **
		Electrode	1	125	0.0475	0.8278
		Duration (check size)	2	125	0.1557	0.8560
		Electrode × check size	1	125	0.4750	0.4920 *
		Duration × electrode (check size)	2	125	0.0608	0.9410
P100 peak time (*n* = 144)	0.58	Check size	1	125	13.1892	0.0004 ***
		Electrode	1	125	0.0021	0.9635
		Duration (check size)	2	125	2.1584	0.1198 *
		Electrode × check size	1	125	0.6178	0.4334 *
		Duration × electrode (check size)	2	125	1.2045	0.3033 *

Note: Alpha level = 0.5. *df_nom_* indicated degrees of freedom numerator. *df_den_* indicates degrees of freedom denominator. Stars indicate the level of significance: * *p* < 0.5, ** *p* < 0.1, *** *p* < 0.01.
